# Near‐Freezing‐Temperature Golgi Neuronal Staining for X‐ray Imaging of Human Brain

**DOI:** 10.1002/advs.202504468

**Published:** 2025-05-28

**Authors:** Feng Zhou, Qiaowei Tang, Xin Yan, Chao Ma, Yu Zhang, Jichao Zhang, Qian Li, Lihua Wang, Jun Hu, Xiaoqing Cai, Jiang Li, Ying Zhu, Chunhai Fan

**Affiliations:** ^1^ CAS Key Laboratory of Interfacial Physics and Technology, Shanghai Institute of Applied Physics Chinese Academy of Sciences Shanghai 201800 China; ^2^ University of Chinese Academy of Sciences Beijing 100049 China; ^3^ Institute of Materiobiology, College of Sciences Shanghai University Shanghai 200444 China; ^4^ Xiangfu Laboratory Jiaxing 314102 China; ^5^ National Human Brain Bank for Development and Function, School of Basic Medicine Peking Union Medical College Institute of Basic Medical Sciences Chinese Academy of Medical Sciences Beijing 100005 China; ^6^ Shanghai Synchrotron Radiation Facility, Shanghai Advanced Research Institute Chinese Academy of Sciences Shanghai 201210 China; ^7^ State Key Laboratory of Synergistic Chem‐Bio Synthesis, School of Chemistry and Chemical Engineering, New Cornerstone Science Laboratory, Frontiers Science Center for Transformative Molecules, Zhang Jiang Institute for Advanced Study and National Center for Translational Medicine Shanghai Jiao Tong University Shanghai 200240 China

**Keywords:** mercury chloride (HgCl_2_), near‐freezing‐temperature (NFT) Golgi, neuron, post‐mortem frozen human brain (PMF‐human brain), synchrotron‐based X‐ray microscopy

## Abstract

Achieving detailed neuronal structural information in large‐volume brain tissue has been a longstanding challenge in human brain imaging. A key obstacle arises from the trade‐off between staining efficiency and tissue autolysis. Traditional Golgi staining, typically conducted at room temperature or 37 °C to optimize staining efficiency, leads to rapid autolysis of brain tissue, resulting in the loss of fine structural details. Here, a near‐freezing temperature (NFT) staining strategy in post‐mortem frozen (PMF) human brain samples are presented, using a mercury chloride‐based method under ice‐water bath conditions. In contrast to the 37 °C Golgi staining, this NFT‐based method significantly reduces tissue autolysis, preserving fine neuronal structures. Notably, neuronal counts in the same field of view increased by 5.5‐fold, and dendritic spine density increases by 22‐fold. Using this approach, uniform staining of millimeter‐thick is achieved, centimeter‐scale human brain slices and integrated it with synchrotron‐based X‐ray microscopy to perform micrometer resolution 3D reconstructions of the cerebellum and frontal lobe. This novel technique offers a powerful tool for the fine‐structural imaging of large‐volume brain tissue, providing new insights into the intricate organization of neural networks.

## Graphical abstract

A near‐freezing temperature staining strategy, combined with a mercury chloride‐based method, effectively preserves delicate neuronal structures in post‐mortem frozen human brain tissues by minimizing autolysis. This approach enables uniform labeling across centimeter‐scale slices and, when integrated with synchrotron‐based X‐ray microscopy, achieves micrometer‐resolution 3D reconstructions, paving the way for large‐scale human brain mapping.

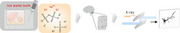

## 1 Introduction

The human brain, as the most complex organ in the body, is central to understanding brain function, development, and advancing the diagnosis and treatment of neurological diseases.^[^ ^1^ ^]^ For instance, Bethlehem et al. utilized magnetic resonance imaging to identify structural abnormalities in the brains of Alzheimer's disease patients, offering vital insights for early diagnosis.^[^ ^2^ ^]^ Segal et al. integrated lesion network mapping with normative modeling to explore individual heterogeneity in mental disorders, spanning from localized brain regions to global networks.^[^ ^3^ ^]^ In mesoscale research, Shan et al. used confocal fluorescence microscopy to image single‐cell morphology in 300 µm thick human temporal lobe tissue, enhancing our understanding of brain structure and function.^[^ ^4^ ^]^ The development of high‐resolution imaging techniques capable of balancing large‐volume sample analysis with fine neuronal structure observation is essential for these studies, providing critical data for cross‐scale brain science.^[^ ^5^ ^]^

Synchrotron‐based X‐ray microscopy offers unique advantages in brain imaging.^[^ ^6^ ^]^ First, X‐rays have high penetrating power, making it possible to image large, especially thick samples.^[^ ^7^ ^]^ For example, Heinzer et al. imaged a 1 cm thick whole mouse brain vasculature with a pixel resolution of 16 µm,^[^ ^8^ ^]^ and Fonseca et al. imaged a 1 cm thick mouse brain at a pixel resolution of 4 µm.^[^ ^9^ ^]^ Similarly, we imaged mouse brain neurons at a voxel resolution of 3.25 µm.^[^ ^10^ ^]^ In contrast, techniques such as micro‐optical sectioning tomography and electron microscopy (EM), which require the preparation of numerous thin sections, often complicate sample preparation, risk tissue damage, and may lead to the loss of crucial information.^[^ ^1^, ^11^ ^]^ Synchrotron‐based X‐ray microscopy reduces the need for sectioning, streamlining tissue preparation and improving imaging efficiency.^[^ ^12^ ^]^ Additionally, the short wavelength of X‐rays provides sub‐micron and even nanometer spatial resolution, ideal for imaging the fine structure of brain tissue.^[^ ^13^ ^]^ While light sheet fluorescence microscopy can achieve similar high‐resolution imaging, it typically necessitates lengthy tissue clearing processes.^[^ ^14^ ^]^ Moreover, X‐rays have excellent elemental resolution, allowing for the detection of high atomic number elements,^[^ ^15^ ^]^ which, when combined with specific element‐based staining techniques, can facilitate high‐resolution imaging of brain structures.^[^ ^12^, ^13^ ^]^ The high brightness and coherence of synchrotron radiation further enhance signal‐to‐noise ratios and imaging sensitivity, offering invaluable support for the in‐depth analysis of brain neural networks.^[^ ^5^ ^]^

A significant challenge in applying synchrotron‐based X‐ray microscopy to human brain tissue lies in the low natural contrast of neurons,^[^ ^16^ ^]^ necessitating the development of effective staining or labeling methods. The Golgi staining technique, which randomly labels neurons with elements such as mercury and silver, produces contrast suitable for X‐ray and EM, making it an indispensable tool for studying neuronal morphology.^[^ ^17^ ^]^ However, the trade‐off between staining efficiency and tissue autolysis remains a limitation, as freshly obtained human brain tissue^[^ ^4^, ^18^ ^]^ is difficult to obtain and degrades rapidly.^[^ ^19^ ^]^ Classical Golgi staining, performed at 37 °C or room temperature to enhance dye diffusion, leads to rapid tissue autolysis, damaging fine structures.^[^ ^20^ ^]^ Freezing techniques can mitigate this autolysis by slowing metabolic processes, inhibiting autolysis and preserving neuronal structure.^[^ ^21^ ^]^ Thus, there is a need for an efficient staining method that is compatible with low‐temperature conditions, enhancing dye penetration in large samples while maintaining neuronal integrity.

**Scheme 1:**
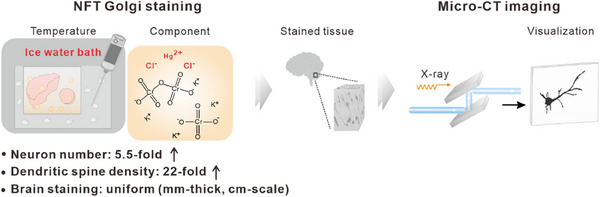
Schematic diagram of the NFT Golgi staining method. PMF human brain tissue was stained for 30 days using the NFT Golgi staining method and subsequently imaged with the synchrotron‐based X‐ray micro‐computed tomography (Micro‐CT).

Drawing inspiration from the use of near‐freezing temperature (NFT) in organ preservation, which lowers metabolic activity and inhibits microbial growth and oxidative enzyme activity without damaging tissue,^[^ ^22^ ^]^ we discovered a mercury chloride‐based Golgi staining method. This approach efficiently stains 3 mm thick post‐mortem frozen (PMF) human brain samples under NFT conditions, achieved through ice‐water bath treatment. This method significantly inhibits tissue autolysis and enhances staining quality. Combined with synchrotron‐based X‐ray microscopy, we successfully achieved micrometer resolution 3D imaging of the human cerebellum and frontal lobe (**Scheme** **1**).

## 2 Results

### 2.1 Establishment of NFT Golgi Neuronal Staining Method for PMF‐Human Brain

**Figure 1:**
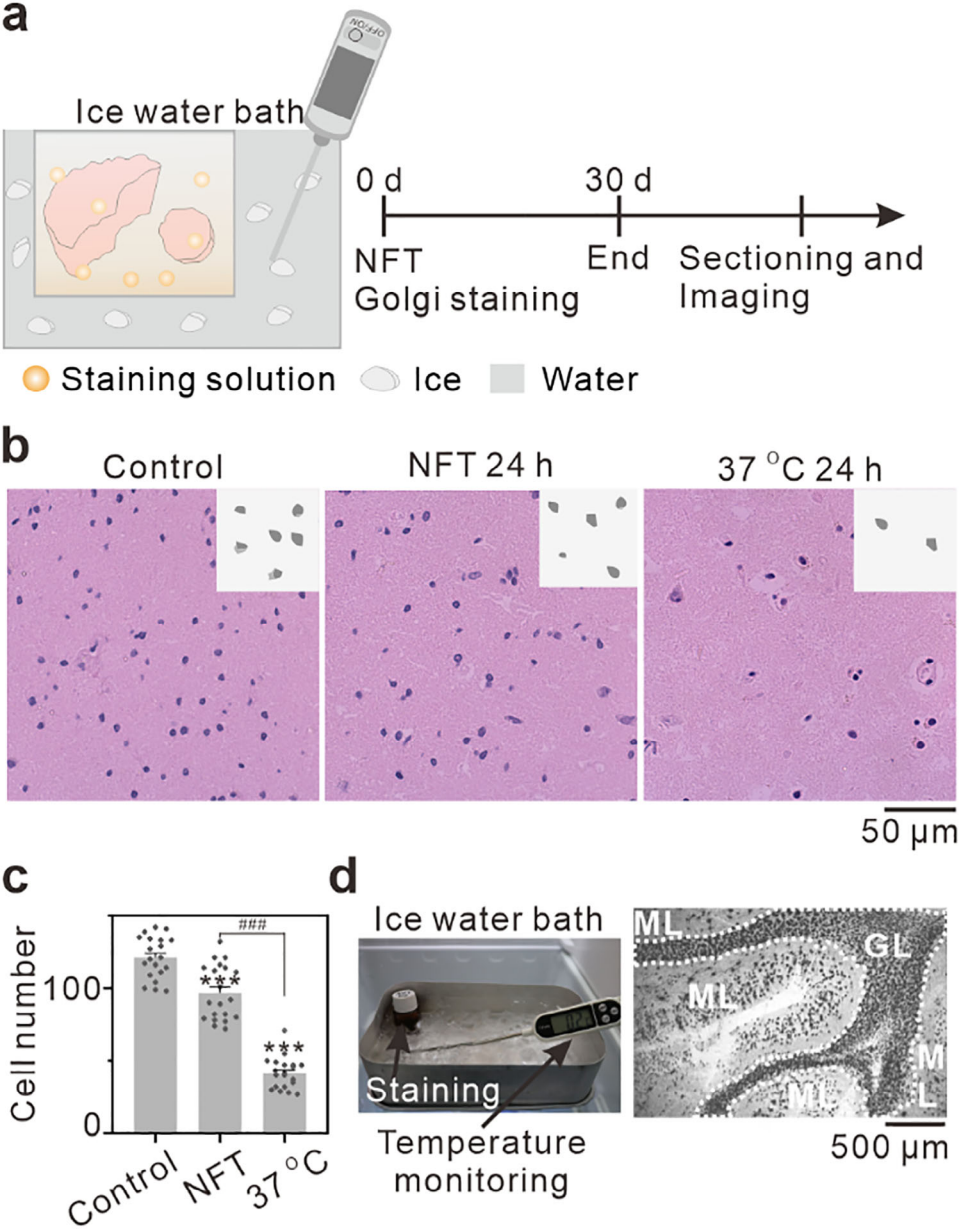
Establishment of the NFT Golgi neuronal staining method for PMF‐human brain. a) Schematic diagram of NFT Golgi staining, human brain tissue was stained for 30 days in an ice water bath device. b) H&E staining results of human brain under different conditions. From left to right: freshly thawed brain tissue, placed at NFT for 24 hours and at 37 °C for 24 hours. Scale bar: 50 µm. c) Cell number statistics of H&E staining results under different conditions, grouped as in b. Data are presented as mean ± SEM (columns with error bars) for n  =  20 independent samples. ***p < 0.001 by t‐test, significantly different from Control group; ^###^ *p* < 0.001 by one‐way analysis of variance (one‐way ANOVA), significantly different from NFT group. d) Golgi staining results of neurons stained at NFT. Left: the image of the human brain during staining, where the bottle containing staining solution and human brain was placed in an ice water bath device, right: optical microscope imaging results of human brain tissue after staining. Irregular dashed lines in the images highlight the GL, while the area outside these lines corresponds to the ML, GL: granular layer; ML: molecular layer. Scale bar: 500 µm.

During the method development, we observed that classical Golgi staining was typically performed at room temperature or 37 °C. However, staining PMF human brain tissue at these temperatures led to rapid autolysis and decreased neuronal structural integrity. To confirm this phenomenon, we incubated PMF brain tissue at 37 °C for 24 hours, followed by hematoxylin and eosin (H&E) staining. The results revealed a significant reduction in nuclear density (purple) compared to freshly thawed tissue, likely due to temperature‐induced autolysis (**Figure** **1**b). In contrast, the tissue placed at NFT (an ice‐water bath) for 24 hours exhibited clear nuclei (purple) and cytoplasm (pink) with uniform morphology (Figure 1b). Quantitative analysis showed mean cell numbers of 121, 97, and 41 for the control, NFT, and 37 °C groups, respectively (Figure 1c). Notably, the NFT group exhibited only a 20% reduction in nuclear count compared to the control. These findings demonstrate that the NFT condition effectively mitigates autolysis in human brain tissue, thereby preserving structural integrity. To further evaluate the impact of postmortem delay (PMD) on neuronal autolysis, we conducted H&E staining on human brain tissues with PMD of 5‐ and 20‐ hours. The results revealed clear nuclear and cytoplasmic structures, indicating that the brain tissue maintained good morphology. Further nuclear counts showed that the average number of nuclei in both groups was 118 and 114, respectively, with no significant difference between them (Figure S1, Supporting Information). This suggests that neuronal autolysis occurs at a slower rate under in vivo condition, likely due to stable physiological factors.^[^ ^23^ ^]^

Based on these observations, we propose to perform Golgi staining under the NFT condition and evaluate its impact on tissue preservation and staining efficacy. First, we immersed a 3‐mm‐thick PMF human cerebellar tissue in Golgi staining solution maintained in an ice‐water bath (NFT) for 30 days (Figure 1a,d). The Golgi staining method is a critical technique for studying neuronal morphology. The staining results revealed well‐preserved structural integrity under the NFT condition, including distinct cerebellar granule layers and molecular layers (Figure 1d). This approach provides a potential strategy for Golgi staining of PMF human brain tissue. To further validate NFT efficacy, we performed Nissl staining (a standard neuroanatomical method) at NFT.^[^ ^24^ ^]^ Optical microscopy results clearly showed the cerebellar granule layer and molecular layer structures (Figure S2, Supporting Information). These findings demonstrate that NFT‐based staining effectively preserves neuronal structures, offering an approach for morphological analysis of PMF human brain tissue.

### 2.2 Mechanism Analysis of NFT Golgi Neuronal Staining

**Figure 2:**
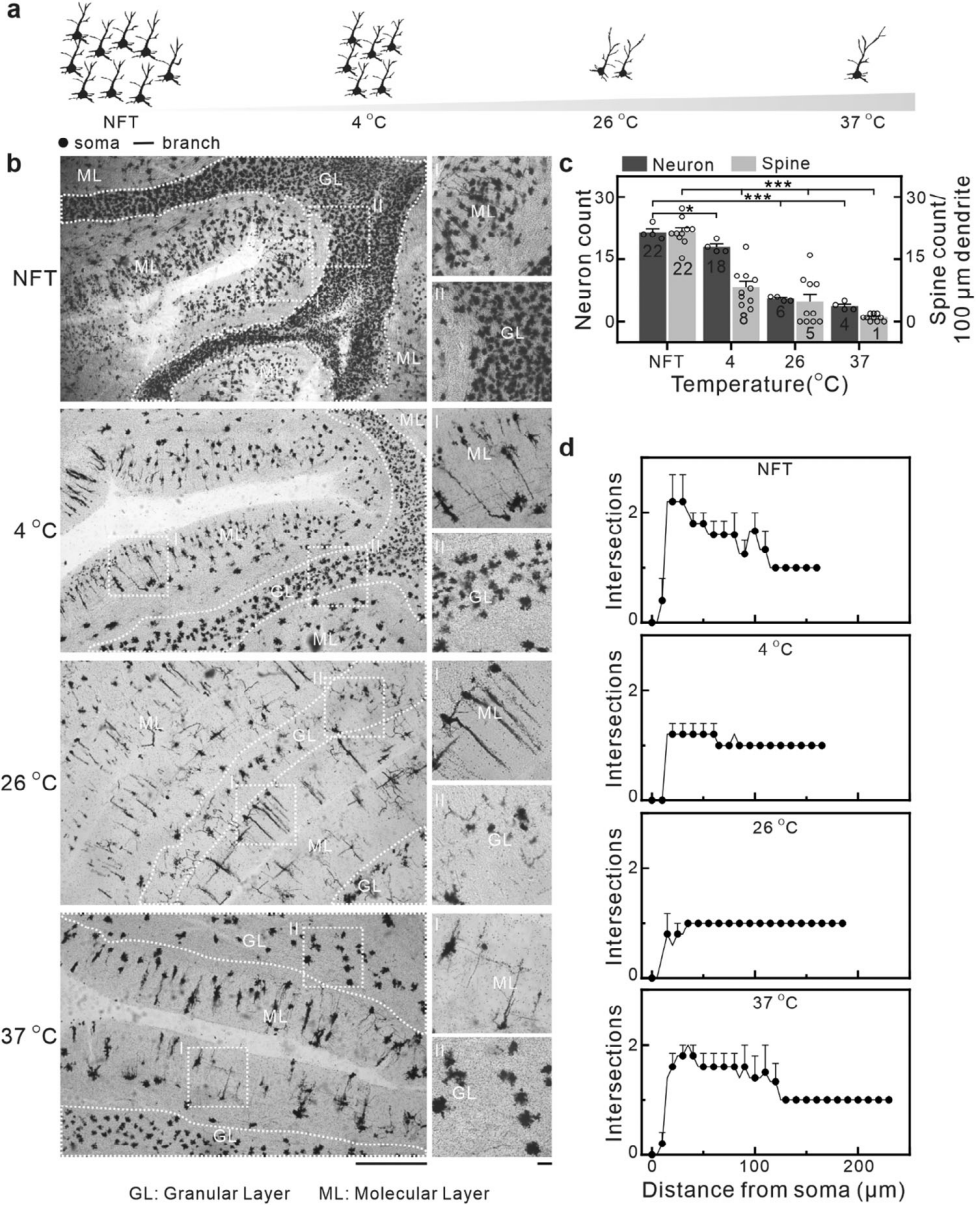
Mechanism analysis of NFT Golgi neuronal staining. a) Schematic representation illustrating neuronal count variation with temperature, showing a gradual increase in neuronal count as the temperature decreases. b) Optical microscope images demonstrating the impact of different temperatures on neuronal staining. Left: low magnification images, right: high magnification images of ML and GL. From top to bottom: NFT, 4 °C, 26 °C, and 37 °C. Irregular dashed lines in the images highlight the GL, while the area outside these lines corresponds to the ML. Scale bar: 500 µm for the left and 50 µm for the right. c) Temperature‐neuron/spine count histograms. Data are presented as mean ± SEM (columns with error bars) for n  =  4 independent samples (neuronal count)/ n  =  10 independent samples (spine count). *p < 0.05; ***p < 0.001, significantly different from NFT group (t‐test). d) Sholl analysis based on the optical microscopy results shown in b, Data are shown as mean ± SEM (dots with error bars) for n  =  5 independent samples.

To investigate the mechanism by which NFT affects Golgi staining in PMF human brain tissue, we systematically evaluated the impact of staining temperature (**Figure** **2**a). PMF human brain tissue was stained under four temperature conditions (NFT, 4 °C, 26 °C, 37 °C) for 30 days in the dark, followed by sectioning (100 µm thickness) and imaging under optical microscopy. Neuronal structures were observed across all conditions, with minimal vascular staining observed (Figure 2b). Quantitative analysis showed mean neuronal soma counts of 22, 18, 6, and 4 per field of view in the NFT, 4 °C, 26 °C, and 37 °C groups, respectively (Figure 2c, Table S1, Supporting Information). Compared to the 37 °C group, the NFT group exhibited a 5.5‐fold increase in neuronal soma density. Dendritic spine density analysis revealed that, in the 37 °C group, only one stained dendritic spine was observed per 100 µm of dendrite, whereas in the NFT group, this number increased to about 22‐fold enhancement (Figure 2c, Table S2, Supporting Information). These results suggest that higher staining temperatures correlate with reduced neuronal soma counts and dendritic spine density, suggesting lower‐temperature conditions (such as NFT) may better preserve tissue integrity and enhance visualization of fine structures. This may be attributed to suppressed autolysis at lower temperatures, potentially preserving neuronal ultrastructure. Furthermore, Sholl analysis of cerebellar neurons across different staining temperatures (Figure 2d) revealed that the longest observable neuronal lengths were 160 µm, 165 µm, 185 µm, and 230 µm for the NFT, 4 °C, 26 °C, and 37 °C groups, respectively, suggesting an inverse correlation between temperature and maximum observable neuronal length, potentially due to temperature‐dependent staining kinetics. However, the NFT group exhibited a pronounced increase in proximal (20–30 µm) intersection counts, suggesting superior preservation of proximal neuronal architecture under low‐temperature conditions. In addition, we incorporated the Hito Golgi‐Cox OptimStain Kit, a commercially available reagent based on the Golgi‐Cox method. Experiments were conducted under both 37 °C and NFT conditions, with subsequent statistical analysis on neuronal count, dendritic spine density, and neuronal complexity. The results show that under NFT condition, stained neuron count and dendritic spine density increased significantly—by approximately 4.2‐fold and 10.5‐fold, respectively. Furthermore, neurons exhibited higher neuronal complexity under NFT condition. The above results further verify the superiority of NFT condition in neuronal Golgi staining (Figure S3, Supporting Information).

To explore the potential mechanisms underlying the enhanced effectiveness of NFT condition compared to room temperature or 37 °C, we conducted biochemical analyses focusing on tissue autolysis. Specifically, we measured levels of trimethylamine (TMA), a simple volatile amine that serves as a marker for tissue autolysis and decay.^[^ ^25^ ^]^ Using Sprague Dawley rat brain tissue as the model, we compared TMA concentrations across different conditions, including freshly isolated brain (Control), isolated brain with the PMD time of 6 hours (0 h group), and varying temperatures (NFT condition vs 37 °C) and in vitro time (2, 6, 12, and 24 h, all with a fixed PMD time of 6 h). The results showed that TMA levels in the Control and 0 h groups were 1.9 mg L^−1^ and 1.8 mg L^−1^, respectively. Although TMA levels remained below 2 mg L^−1^ at 2 and 6 h under 37 °C condition, a significant increase to 15.7 mg L^−1^ was observed at 12 hours. At 24 hours, TMA levels sharply rose to 45.5 mg L^−1^, indicating extensive autolysis of brain tissue occurring. In contrast, under NFT condition, TMA levels in brain tissue remained below 2 mg L^−1^ across all tested in vitro time (Figure S4, Supporting Information). These findings suggest that NFT condition helps preserve tissue integrity by suppressing autolysis processes, thereby contributing to improved staining outcomes.

### 2.3 Effects of Staining Solution Composition on the Quality of Human Brain Neuronal Staining

Having demonstrated the efficacy of NFT conditions in enhancing Golgi staining of PMF human brain neurons, we further investigated the impact of Golgi staining solution composition on staining efficacy. Commonly used Golgi staining formulations primarily fall into four categories, which include (1) potassium dichromate and silver nitrate solutions,^[^ ^26^ ^]^ (2) osmium tetroxide combined with potassium dichromate and silver nitrate solutions,^[^ ^27^ ^]^ (3) glutaraldehyde combined with potassium dichromate and silver nitrate solutions,^[^ ^28^ ^]^ and (4) potassium dichromate, potassium chromate, and mercuric chloride solutions.^[^ ^29^ ^]^ Among these components, mercuric chloride is thought to enhance staining by increasing binding affinity to specific cellular components, such as proteins.^[^ ^30^ ^]^ Silver nitrate plays a crucial role in neuronal morphology studies by reacting with potassium dichromate to form silver chromate precipitates.^[^ ^31^ ^]^ Osmium tetroxide and potassium dichromate may contribute to stabilizing neuronal structure and reducing nonspecific staining, thereby improving the visualization of neuronal morphology.^[^ ^32^ ^]^ Optimizing the combination of these components is essential for enhancing staining quality, providing higher‐quality data for neuronal morphological studies.

**Figure 3:**
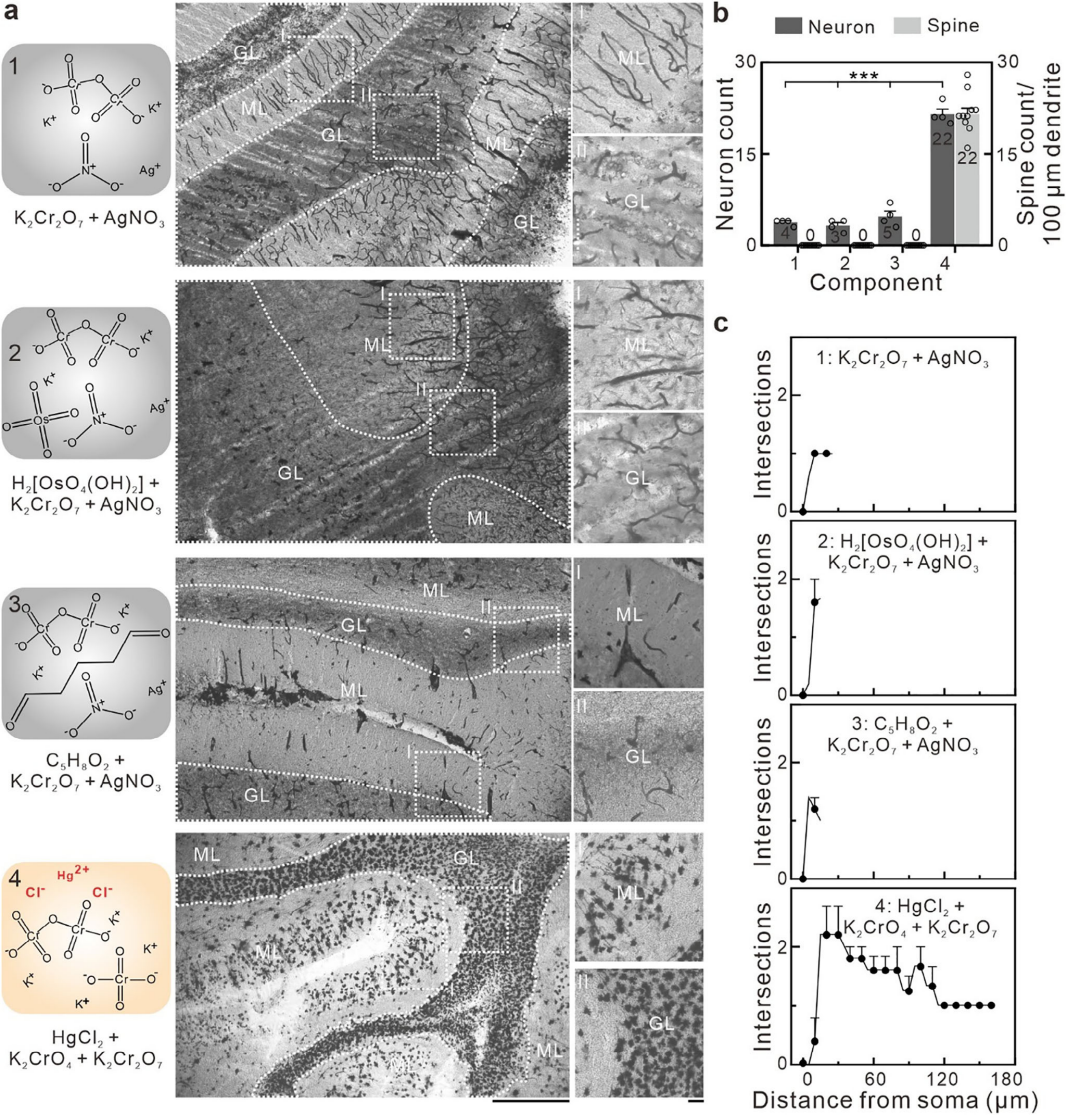
Effect of staining solution components on the structure of stained PMF‐Human brain neurons. a) Optical microscopy images demonstrating the impact of different staining solution components on neuronal structure, left: chemical structure formula of components in each staining solution, middle: low magnification images, right: high magnification images of ML and GL. 1: potassium dichromate and silver nitrate; 2: potassium dichromate, osmium tetroxide, and silver nitrate; 3: glutaraldehyde, potassium dichromate, and silver nitrate; 4: potassium dichromate, potassium chromate, and silver nitrate. Irregular dashed lines in the images highlight the GL, while the area outside these lines corresponds to the ML. Scale bar: 500 µm for the left and 50 µm for the right. b) Component‐neuron/spine count histograms. Data are presented as mean ± SEM (columns with error bars) for n  =  4 independent samples (neuronal count)/n  =  10 independent samples (spine count). ***p < 0.001, significantly different from composition 4 group (one‐way ANOVA). c) Sholl analysis based on the optical microscopy results shown in b, Data are shown as mean ± SEM (dots with error bars) for n  =  5 independent samples.

Under the NFT condition, PMF human cerebellar tissues were stained for 30 days, sectioned (100 µm thickness), and imaged under optical microscopy. Staining with compositions 1–3 resulted in predominant vascular labeling and sparse neuronal staining (**Figure** **3**a). In contrast, composition 4 produced dense neuronal soma labeling with minimal vascular interference, demonstrating a significantly improved neuron‐to‐blood vessel staining ratio. Furthermore, high‐magnification images clearly showed well‐preserved dendritic spines. Additionally, compared with the other compositions, composition 4 enabled clear distinction between the granule layers and molecular layers of the cerebellum.

Quantitative analyses further supported these observations. Neuronal soma count analysis revealed mean values of 4, 3, and 5 somata per field of view for compositions 1, 2, and 3, respectively, whereas composition 4 yielded a significantly higher count of 22 (Figure 3b, Table S3, Supporting Information). Dendritic spine density analysis showed no detectable spines in compositions 1–3, while composition 4 achieved a density of 22 spines per 100 µm dendrite (Figure 3b, Table S4, Supporting Information). Sholl analysis further indicated that composition 4 enabled the longest observable dendritic length (160 µm) and the highest number of proximal intersections (20–30 µm from the soma), representing 6.4‐, 10.7‐, and 10.7‐fold improvements over compositions 1, 2, and 3, respectively (Figure 3c). This indicates that the neurons stained with composition 4 provided the highest neuronal structural integrity.

Additionally, in conventional Golgi staining protocols, the staining time is typically recommended to be 2 weeks for optimal visualization,^[^ ^33^ ^]^ while extended time beyond one month should be avoided to prevent non‐specific staining.^[^ ^10^, ^34^ ^]^ We conducted short‐duration (15‐day) Golgi staining under NFT condition followed by neuron count statistics, spine density statistics and Sholl analysis. Although there were non‐significant differences in neuron counts between the two groups, the 30‐day staining exhibited a significant increase in spine density. Moreover, the results of Sholl analysis demonstrated the higher neuronal complexity in 30‐day staining, indicating more complete staining (Figure S5, Supporting Information). Simultaneously, 30‐day staining exhibited minimal nonspecific staining compared to 15‐day group. Based on the references and experimental results, we set the staining time as 30 days.

Above all, under the NFT condition, staining PMF human brain tissue with composition 4 for 30 days not only significantly increases the number of stained neurons but also preserves their structural integrity, providing a solid foundation of subsequent imaging and analysis.

### 2.4 Synchrotron‐Based X‐ray Micro‐CT Imaging of PMF‐Human Brain Neurons

**Figure 4:**
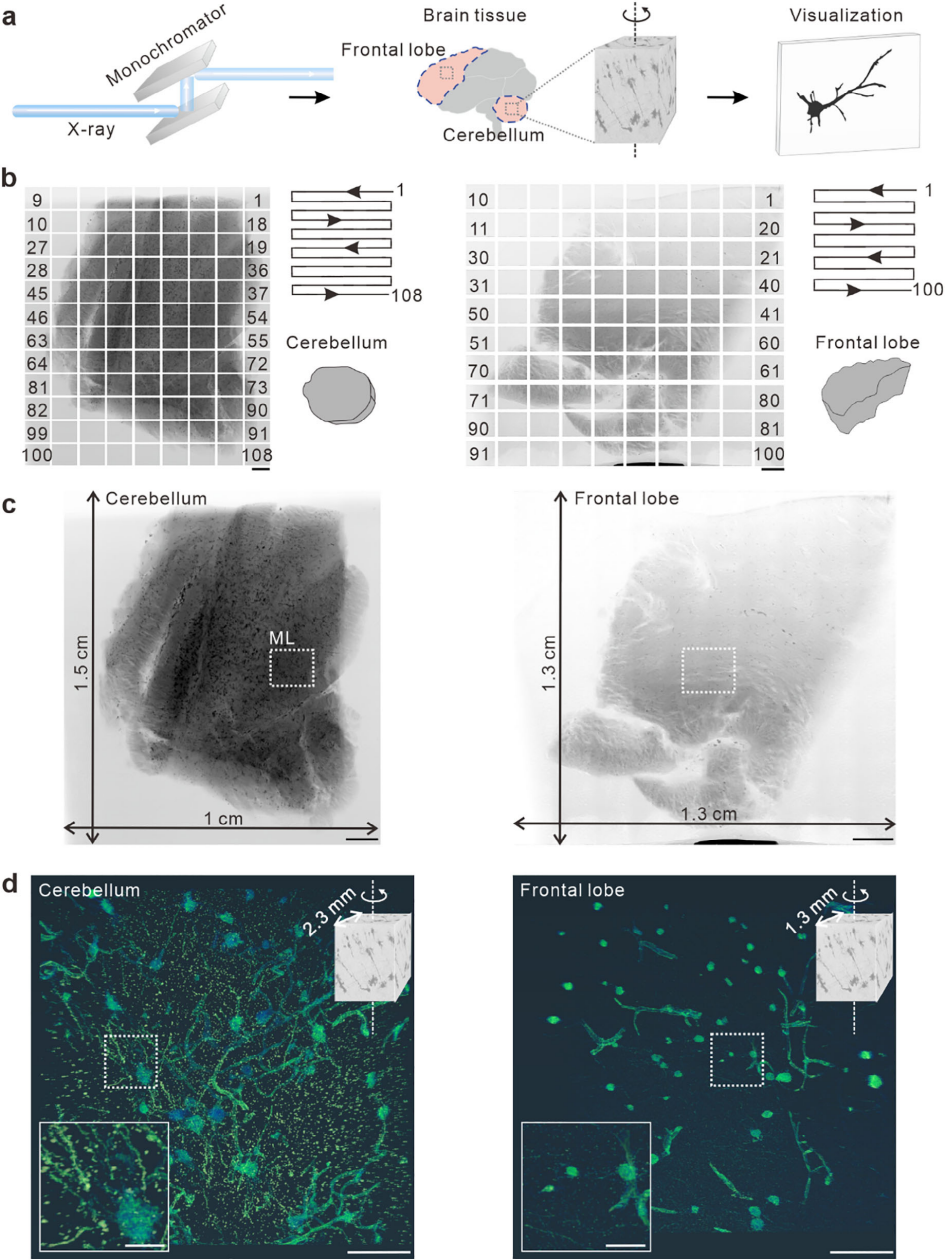
Synchrotron‐based X‐ray Micro‐CT imaging of PMF‐human cerebellar neurons. a) Schematic of synchrotron‐based X‐ray imaging, illustrating the imaging of neurons in the cerebellum and frontal lobe. b) 2D image stitching process and 2D serpentine stitching schematic, left: cerebellum, right: frontal lobe. Scale bar: 1 mm. c) 2D imaging results with a large field of view. left: cerebellum, right: frontal lobe. Scale bar: 1 mm. d) High‐resolution 3D visualization of the boxed area in c and high magnification images. left: cerebellum, right: frontal lobe. Scale bar: 200 µm for 3D visualization and 50 µm for high magnification images.

To analyze neuronal morphology in 3D, we selected millimeter‐thick, centimeter‐scale cerebellar and frontal lobe tissue sections of PMF human brain, stained them using the NFT Golgi method, and performed synchrotron‐based X‐ray Micro‐CT imaging after 30 days of staining (**Figure** **4**a). First, we conducted large‐field‐of‐view 2D imaging at the centimeter scale to assess the overall morphology of the samples. Imaging was performed at a pixel resolution of 0.5 µm with a 20% overlap in both horizontal and vertical directions. A “Grid: snake by rows” scanning and stitching strategy.^[^ ^35^ ^]^ was employed, capturing a total of 108 and 100 projection images for the cerebellum and frontal lobe, respectively (Figure 4b). The stitched images revealed the morphology of the sections, especially for the cerebellum, where distinct brain regions and neuronal soma were clearly observed (Figure 4c). These results demonstrate that NFT Golgi‐stained human brain tissue is suitable for synchrotron‐based X‐ray imaging.

Next, we performed 3D imaging of NFT Golgi‐stained human brain tissue using synchrotron‐based X‐ray Micro‐CT. We selected specific regions within the above stitched images for high‐resolution 3D imaging at the cubic‐millimeter scale. Figure S6 (Supporting Information) presents 2D projection images of human cerebellar and frontal lobe tissues of different angles. Neuronal structures were clearly observed, particularly within the 55°–115° range for the cerebellum and the 25°–145° range for the frontal lobe. Subsequently, slice reconstruction and 3D visualization were performed. The results revealed clear neuronal structures in both cerebellar and frontal lobe tissues, with more observed neurons in the cerebellum, which may be due to the high neuronal density of cerebellum (Figure 4d). However, some vascular staining was also observed in both, which may be attributed to the absence of perfusion, resulting in residual blood within the brain tissue. Further optimization of blood clearance techniques will be necessary to improve the specificity of neuronal staining in 3D imaging. Therefore, further optimization of residual blood clearance techniques in human brain tissue is required, such as pre‐chilled (4 °C) PBS/saline, coupled with cyclic immersion and low‐speed agitation under NFT.

These results demonstrate that the NFT Golgi staining method is an effective technique for synchrotron‐based X‐ray Micro‐CT imaging of human brain tissue. By integrating these two approaches, we successfully achieved neuronal staining and high‐resolution 3D imaging across different brain regions. This technique combines the high resolution and rapid imaging capabilities of synchrotron‐based X‐ray Micro‐CT, laying a solid foundation for whole‐brain neuronal staining and neuronal morphology analysis in future studies.

## 3 Conclusion and Discussion

In summary, we discovered a NFT staining strategy that enables efficient labeling of PMF human brain samples using a mercury chloride‐based Golgi method. Compared to conventional Golgi staining at 37 °C, this method significantly improves neuronal visualization, increasing the number of labeled neurons by 5.5‐fold and dendritic spine density by 22‐fold within the same field of view. When integrated with synchrotron‐based X‐ray microscopy, this approach successfully achieved micrometer resolution 3D reconstruction of mm‐thick human cerebellar and frontal lobe tissues.

Compared with emerging high‐resolution imaging techniques, the NFT Golgi staining method offers distinct advantages in visualizing the complete morphology of individual neurons without the need for genetic modification or molecular labeling. While transgenic fluorescent labeling and viral tracing provide cell‐type specificity and are well‐suited for functional studies, they often require complex preparation.^[^ ^36^ ^]^ Tissue clearing combined with light‐sheet microscopy enables large‐scale 3D imaging but depends on fluorescent labeling and may have limited resolution for fine structures.^[^ ^37^ ^]^ Super‐resolution microscopy achieves nanoscale resolution but is constrained by imaging depth and field of view.^[^ ^38^ ^]^ In this context, NFT Golgi staining serves as a complementary tool, providing a simple, unbiased, and efficient approach for neuronal structural analysis.

NFT has been widely applied in the preservation of various biological tissues and organs due to its ability to slow autolysis and maintain cellular integrity.^[^ ^22^, ^39^ ^]^ Under NFT conditions, tissue decomposition is effectively suppressed, thereby preserving fine neuronal structures, making it a promising strategy for both structural preservation and staining. In Golgi staining, mercury chloride enhances neuronal visibility by interacting with intracellular proteins and lipids. Its primary mechanisms include (1) binding to thiol (‐SH) groups to form insoluble precipitates, which enhances structural contrast, (2) stabilizing cellular proteins and lipids to maintain neuronal morphology, and (3) increasing contrast to highlight neuronal details. These effects are mainly attributed to the stable interactions between mercury chloride and sulfhydryl groups in proteins.^[^ ^30^, ^40^ ^]^

Despite its advantages, this technique presents certain limitations that require further optimization. The significantly reduced staining temperature slows down reagent diffusion and chemical reactions, leading to prolonged staining durations and limiting its scalability for high‐throughput applications. Additionally, the reduced staining range in some neurons at low temperatures may hinder the comprehensive visualization of complex neuronal morphology and fine structures. Future research should focus on several key aspects, including (1) investigating the preservation dynamics and enzymatic activity under NFT conditions to better understand tissue autolysis kinetics, (2) optimizing staining formulations and protocols by incorporating rapid‐permeation reagents to enhance staining efficiency and coverage, and (3) developing innovative strategies to further inhibit tissue autolysis while maintaining neuronal morphology. Addressing these challenges will enhance the efficiency and applicability of mercury chloride‐based Golgi staining, providing a more reliable and effective tool for human brain neuronal research. This advancement will facilitate its broader application in both fundamental neuroscience and clinical investigations.

## 4 Experimental Section

### Human Brain Sample Source

Post‐mortem human brain samples from individuals without neurological diseases were provided by the Human Tissue Bank of Peking Union Medical College, Chinese Academy of Medical Sciences and the Fudan Branch of the National Health and Disease Human Brain Tissue Resource Center (Table S5, Supporting Information). All brain tissue samples were conducted using H&E staining, revealing uniform nuclear and cytoplasmic morphology, indicating well‐preserved tissue with no discernible histopathological abnormalities (Figure S7, Supporting Information). All sample collection and experimental procedures followed national laws and international ethical and technical guidelines. The present study was approved by the Institutional Review Board of the Institute of Basic Medical Sciences, Chinese Academy of Medical Sciences (Approval Number: 009–2014, 031–2017, and 2022125) and Ethics Committee of Shanghai University (Approval Number: ECSHU 2024–141). After snap‐freezing with liquid nitrogen, brain samples were stored at −80 °C before processing. Frozen human brain tissue was used for subsequent staining experiments. For the experiments of temperature and staining solution components on neuronal staining and so on in this study, the same brain region and the same depth position from the same sample was used to reduce the bias.

### H&E Staining

Frozen brain tissues were thawed at room temperature, and the brain tissues were divided into Control group, NFT group and 37 °C group, respectively. The brain tissues in Control group were directly to 4% paraformaldehyde, and the brain tissues in the NFT group and 37 °C group were placed in saline for 24 hours, respectively, and then preserved using 4% paraformaldehyde. Brain tissues were subjected to tissue dehydration, tissue clear, dipping paraffin, embedding, sectioning and baking, after the sections were deparaffinized. Sections were stained into Harris' hematoxylin stain for 5–7 minutes, and returned to blue by water immersion, then the sections were differentiated into 1% hydrochloric acid in alcohol for 2–5 seconds, and returned to blue by water immersion, and then stained into 1% water‐soluble eosin stain for 2 minutes, and returned to blue by water immersion for 30 seconds. Finally, sections were dehydrated by immersion in anhydrous ethanol, cleared with xylene, air‐dried and sealed with neutral gum, and imaged using optical microscopy.

### Effect of Temperature on Staining PMF‐Human Brain Neurons

PMF‐human brain tissues were thawed at room temperature and trimmed to a thickness of 3 mm. The tissues were then immersed in a staining solution containing 1% potassium dichromate, 1% potassium chromate, and 1% mercury chloride. Staining was performed at different temperatures: 37 °C, 26 °C, 4 °C and NFT, away from light. The staining solution was replaced on the 1st, 8th, 15th, 22nd and 28th days with the total staining duration lasting 30 days. Following staining, the tissues underwent 30% sucrose solution dehydration for 3 days, followed by Tissue Tek O.C.T compound embedding, frozen sectioning, and overnight drying. Subsequent dehydration was carried out using graded alcohol (30%, 50%, 70%, 90%, 95%, and 100%), followed by clearing with in xylene. The samples were then mounted with Eukitt (Sigma, 0 3989) and imaged using optical microscopy.

### Effect of Staining Solution Components on Staining PMF‐human Brain Neurons

PMF‐human brain tissues were thawed at room temperature and trimmed to a thickness of 3 mm. The tissues were immersed in staining solutions 1, 2, 3, and 4 at NFT, away from light. Following the staining process, the tissues underwent 30% sucrose solution dehydration for 3 days, followed by Tissue Tek O.C.T. compound embedding, frozen sectioning, and overnight drying. Subsequent dehydration was carried out using graded alcohol series (30%, 50%, 70%, 90%, 95%, and 100%), followed by clearing with xylene. The samples were mounted with Eukitt (Sigma, 0 3989) and imaged using optical microscopy. Details of the staining solutions: Staining Solution 1: Contained potassium dichromate and silver nitrate. The tissues were immersed in 1.25% potassium dichromate for 10 days and then transferred to a 1% silver nitrate solution for 20 days. Staining Solution 2: Comprised potassium dichromate, osmium tetroxide, and silver nitrate. The tissues were immersed in a mixture of 2% potassium dichromate and 0.16% osmium tetroxide for 10 days and then transferred to 1% silver nitrate solution for 20 days. Staining Solution 3: Consisted of glutaraldehyde, potassium dichromate, and silver nitrate. The tissues were immersed in a mixture of 5% glutaraldehyde and 4% potassium dichromate solution for 10 days and then treated with 1% silver nitrate solution for 20 days. Staining Solution 4: Included potassium dichromate, potassium chromate, and mercuric chloride. The tissues were immersed in a mixture of 1% potassium dichromate, 1% potassium chromate, and 1% mercuric chloride for 30 days. The staining solution was replaced with fresh solution on the 1st, 8th, 15th, 22nd, and 28th days.

### Synchrotron‐Based X‐ray Imaging

Synchrotron‐based X‐ray imaging of human brain tissue was performed at the Brain Imaging Beamline (BIB) of the Taiwan Photon Source (TPS) in Taiwan, China. For large‐field‐of‐view 2D imaging, paraffin‐embedded tissue samples were positioned on the sample stage and scanned using an imaging energy of 14 keV. The scanning boundaries were set based on the tissue's length and width prior to imaging, enabling automated sequential imaging in a systematic left‐to‐right and top‐to‐bottom pattern, ensuring full coverage of the tissue block. During image acquisition, 20% overlap was set in either the horizontal or vertical direction between adjacent images to facilitate subsequent software‐based recognition and stitching. After image acquisition, the background was subtracted using the Image Calculator module in Fiji ImageJ, followed by image stitching with the Stitching module.^[^ ^35^ ^]^

The human cerebellar tissue sample measured 1.0 cm × 1.5 cm × 2.3 mm, with a thickness of 2.3 mm, while the human frontal cortex tissue block measured 1.3 cm × 1.3 cm × 1.3 mm, with a thickness of 1.3 mm. Following 2D imaging, specific regions of the tissue block were selected for localized 3D imaging. At 14 keV, the imaging setup rotated each dataset by 180 degrees in 0.3‐degree increments, capturing 601 frames in total. Background images were acquired every 300 frames, with an exposure time of 200 ms per frame. The raw 2D projection images were reconstructed into 3D slices using PITRE software. These slices were analyzed and processed with Fiji ImageJ, and the results were visualized using Amira rendering software. This approach enabled detailed visualization of neuronal structures, offering insights into the morphology and distribution of neural networks at different levels of the brain tissue.

### Statistical Analysis

Statistical significance of the data was determined by t‐test or one‐way ANOVA using SPSS 26, resenting results as mean ± SEM. * euqals *p* < 0.05; ** equals *p* < 0.01; *** equals *p* < 0.001. Details of statistical tests for each graph were explained in figure legends.

## Conflict of Interest

The authors declare no conflict of interest.

## Author Contributions

F.Z., Q.T., and X.Y. contributed equally to this work.

## Supporting information



Supporting Information

## Data Availability

The data that support the findings of this study are available from the corresponding author upon reasonable request.
