# Supplementary material for: Near‐Freezing‐Temperature Golgi Neuronal Staining for X‐ray Imaging of Human Brain
Source: Adv Sci (Weinh). 2025 May 28;12(30):e04468. doi: 10.1002/advs.202504468 (PMC12376569; doi:10.1002/advs.202504468)
Supplement: Supplementary file 1 — Supporting Information [file ADVS-12-e04468-s001.docx]

Supporting Information

Near-freezing-temperature Golgi neuronal staining for X-ray imaging of human brain

Feng Zhou^#^, Qiaowei Tang^#^, Xin Yan^#^, Chao Ma, Yu Zhang, Jichao Zhang, Qian Li, Lihua Wang, Jun Hu*, Xiaoqing Cai*, Jiang Li*, Ying Zhu*, Chunhai Fan*

F. Zhou, X. Yan

CAS Key Laboratory of Interfacial Physics and Technology, Shanghai Institute of Applied Physics, Chinese Academy of Sciences, Shanghai 201800, China, University of Chinese Academy of Sciences, Beijing 100049, China

Q. Tang

Institute of Materiobiology, College of Sciences, Shanghai University, Shanghai 200444, China, Xiangfu Laboratory, Jiaxing 314102, China

C. Ma

National Human Brain Bank for Development and Function, School of Basic Medicine Peking Union Medical College, Institute of Basic Medical Sciences Chinese Academy of Medical Sciences, Beijing 100005, China

Y. Zhang, J. Zhang, X. Cai

Shanghai Synchrotron Radiation Facility, Shanghai Advanced Research Institute, Chinese Academy of Sciences, Shanghai 201210, China

E-mail: caixq@sari.ac.cn

Q. Li, C. Fan

State Key Laboratory of Synergistic Chem-Bio Synthesis, School of Chemistry and Chemical Engineering, New Cornerstone Science Laboratory, Frontiers Science Center for Transformative Molecules and National Center for Translational Medicine, Shanghai Jiao Tong University, Shanghai 200240, China

E-mail: fanchunhai@sjtu.edu.cn

J. Hu, J. Li, Y. Zhu

Institute of Materiobiology, College of Sciences, Shanghai University, Shanghai 200444, China

E-mail: hujun64@shu.edu.cn, lijiang80@shu.edu.cn, zhuying331@shu.edu.cn

**Supporting Text**

*Animals*: Sprague-Dawley (SD) rat (250 ± 20 g, male) were purchased from Shanghai SLAC Laboratory Animal Co., Ltd., China. All the animals experiments were conducted in accordance with the Institute's Guide for the Care and Use of Laboratory Animals and were approved by the ethical committee of Shanghai Beautiful Life Medica, Technology (approval no. SYXK-2017-0016, approved on 25 December 2017).

*Nissl Staining:* Frozen brain sections were thawed at room temperature and trimmed a thickness of 3 mm. Brain tissues were fixed using 4% PFA solution at 4 ℃ overnight, stained with Nissl staining solution at NFT for 30 days, washed, 30% sucrose solution dehydration for 3 days, frozen sectioned, dehydrated using alcohol in gradients (30%, 50%, 70%, 90%, 95%, and 100%), cleared with xylene, mount by Eukitt (Sigma, 03989), and imaged by optical microscopy.

*Sholl Analysis*: Sholl analysis, also known as concentric circle analysis, is a quantitative analysis of neuronal axons and dendrites, which can be used to assess the complexity of neurons, and can be analyzed using Fiji Image J image processing software. The principle is to take the soma as the center, superimpose a set of concentric circles with the same distance interval on the neuron, count the number of intersections between each concentric circle and the neuronal branches, and derive the branching categories of neuronal dendrites and axons, so as to analyze the morphological characteristics of neurons quantitatively, reflecting the complexity of the neuron.

*TMA level measurement*: Brain tissue (1.2 g) was homogenized in 5% Trichloroacetic acid (TCA) (2.4 mL, 4 °C), and this step should be completed as quickly as possible at 4 °C. After centrifugation at 4000 rpm for 5 minutes, 2 mL of the supernatant was transferred to a headspace vial, followed by the addition of 6 mL of 33% NaOH solution. The headspace equilibrium temperature was set to 40 °C and maintained for 30 minutes. The injection port temperature was 210 °C, the ion source temperature was 230 °C, and the column temperature was initially held at 40 °C for 5 minutes, then increased to 210 °C at a rate of 20 °C/min and maintained for 10 minutes. Helium (99.999%) was used as the carrier gas at a flow rate of 1 mL/min. A 0.5 mL gas-phase sample was injected into the headspace gas chromatography (HS-GC). All data were processed using Agilent MassHunter software (Version B.09.00).

*Sample Preparation for Synchrotron-based X-ray Imaging*: Human brain tissues that had completed NFT Golgi staining were subjected to gradient dehydration (30%, 50%, 70%, 90%, 95%, 100%) in alcohol for 12 hours for each process. Subsequently, the tissues were transferred and transferred to a mixture of xylene and alcohol with a ratio of 1:1 to be immersed in xylene at 40 ℃ for 2 hours and replaced with a fresh mixture of the solution at 1-hour intervals, followed by transferring the tissues to xylene at 40 ℃ for immersed for 2 hours, replacing the fresh xylene solution every 1 hour. Then the tissues were immersed in molten paraffin for 2 hours, replacing the fresh molten paraffin solution every 1 hour. Finally, the human brain tissues were embedded using a paraffin embedding machine.


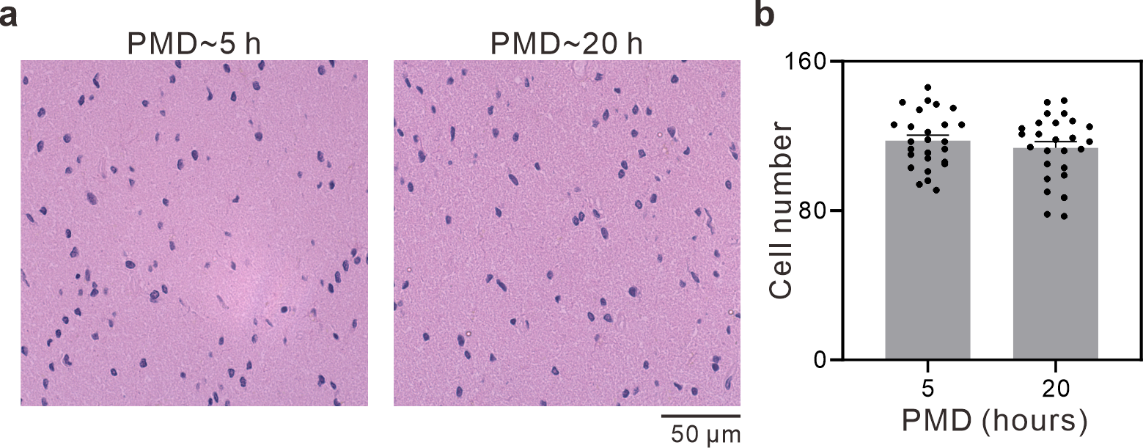


**Figure S1. Effect of PMD time on autolysis of brain tissue. a,** H&E staining results of brain tissue with different PMD time. **b,** PMD time-cell number histograms. Data are presented as mean ± SEM (columns with error bars) for n = 26 independent samples. *p* = 0.386514 by t-test, non-significant different from 20 h.


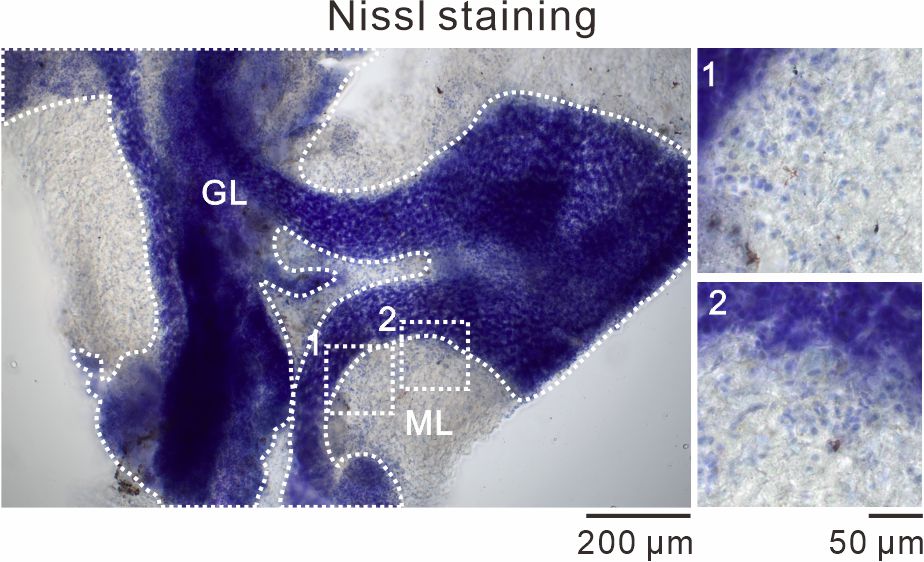


**Figure S2.** **Results of Nissl staining.** Irregular white dotted box is the granular layer and outside the box is the molecular layer. GL: granular layer; ML: molecular layer. Left: image result of low magnification, right: image result of high magnification.

**
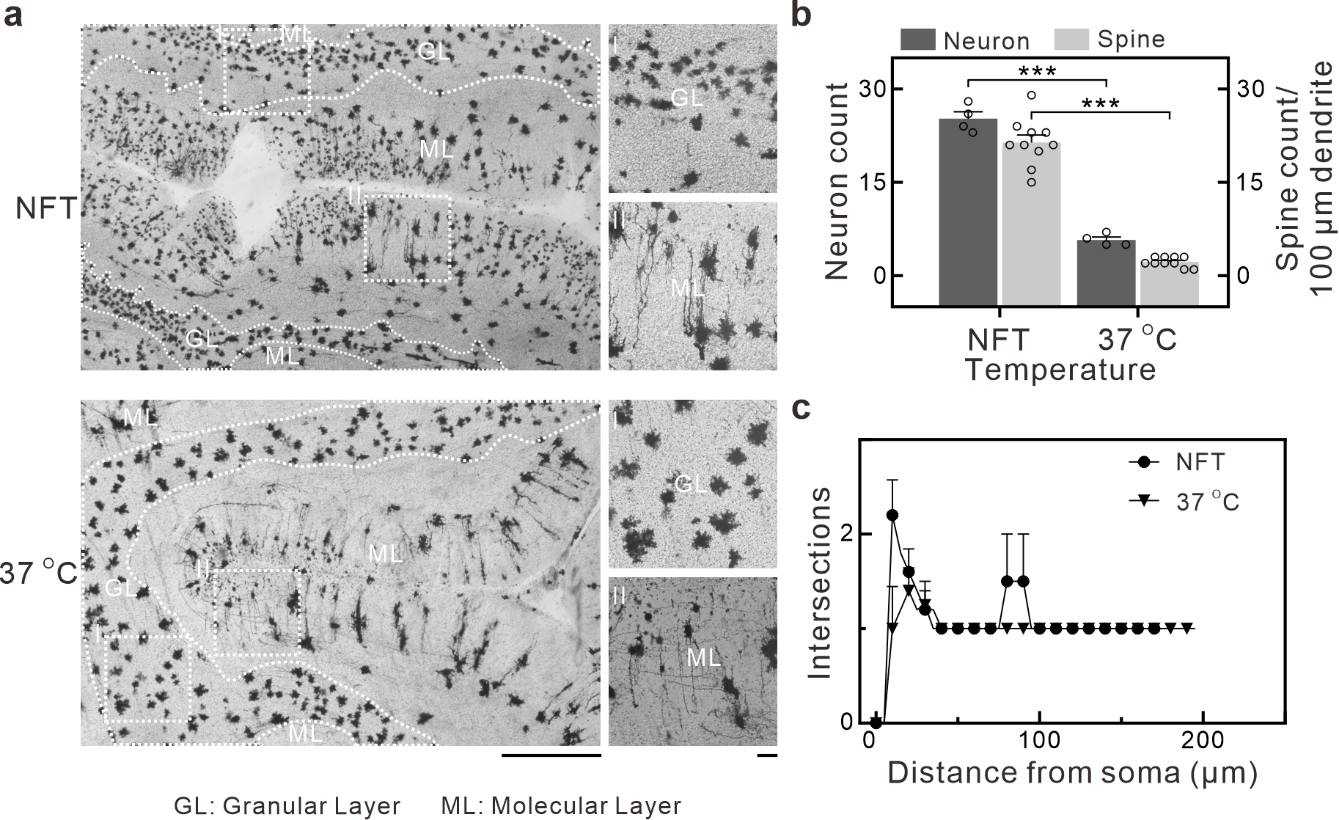
**

**Figure S3. Effect of temperature on neuronal staining via Hito Golgi-Cox OptimStain Kit. a,** Optical microscope images demonstrating the impact of different temperatures on neuronal staining. Left: low magnification images, right: high magnification images of ML and GL. From top to bottom: NFT and 37 °C. Irregular dashed lines in the images highlight the GL, while the area outside these lines corresponds to the ML. Scale bar: 500 μm for the left and 50 μm for the right. **b,** Temperature-neuron/spine count histograms. Data are presented as mean ± SEM (columns with error bars) for n = 4 independent samples (neuronal count)/ n = 10 independent samples (spine count). ****p* < 0.001, significantly different from NFT group (t-test). **c,** Sholl analysis based on the optical microscopy results shown in **a**, Data are shown as mean ± SEM (dots with error bars) for n = 5 independent samples.

**
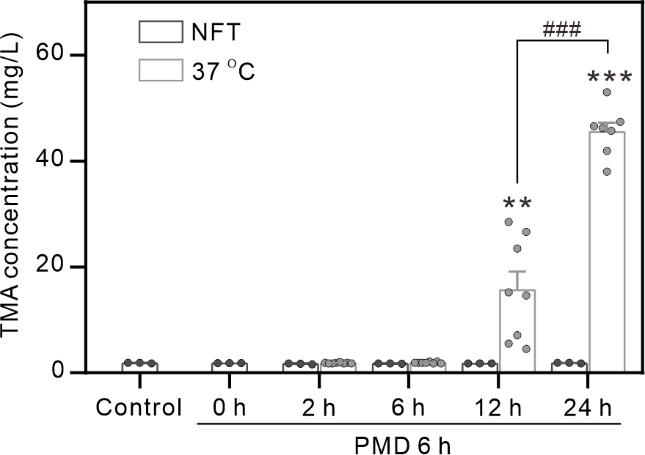
**

**Figure S4. TMA concentrations across different conditions.** Using SD rat brain tissue as the model, Groups were set to freshly isolated brain (Control), isolated brain with the postmortem delay (PMD) time of 6 hours, and varying temperatures (NFT condition vs. 37 °C) and in vitro time (2, 6, 12, and 24 h, all with a fixed PMD time of 6 h). Data are presented as mean ± SEM (columns with error bars) for at least 3 independent samples (n). ***p* < 0.01 (37 °C, 12 h), and ****p* < 0.001 (37 °C, 24 h), significantly different from Control (t-test). ^###^*p* < 0.001 (37 °C, 24 h), significantly different from 12 h group (t-test).

**
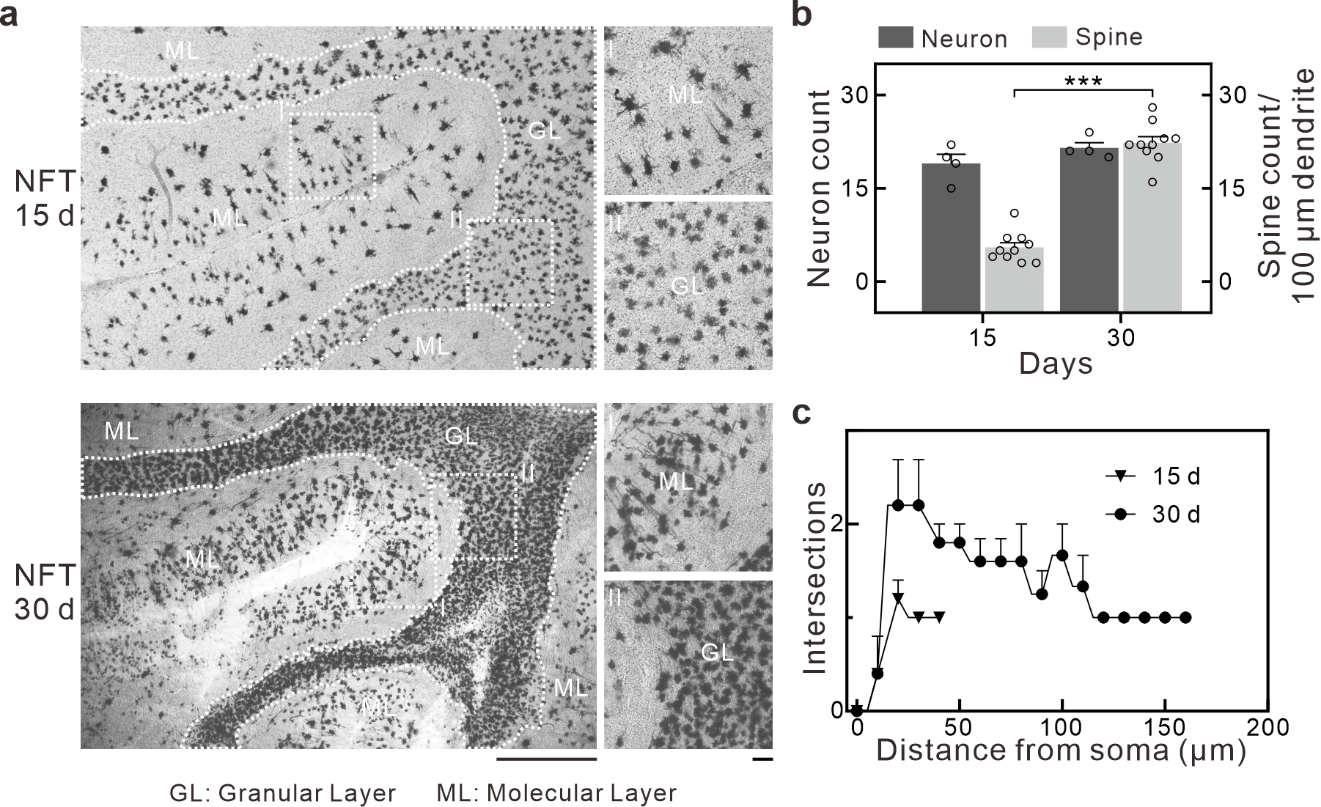
**

**Figure S5. Effect of time on NFT Golgi staining. a,** Optical microscope images demonstrating the impact of different times on neuronal staining. Left: low magnification images, right: high magnification images of ML and GL. From top to bottom: 15 d and 30 d. Irregular dashed lines in the images highlight the GL, while the area outside these lines corresponds to the ML. Scale bar: 500 μm for the left and 50 μm for the right. **b,** Time-neuron/spine count histograms. Data are presented as mean ± SEM (columns with error bars) for n = 4 independent samples (neuronal count)/ n = 10 independent samples (spine count). *p* = 0.193570 by t-test, non-significant difference from 15 d group (neuronal count), ****p* < 0.001 by t-test, significantly different from 15 d group (spine count); **c,** Sholl analysis based on the optical microscopy results shown in **a**, Data are shown as mean ± SEM (dots with error bars) for n = 5 independent samples.


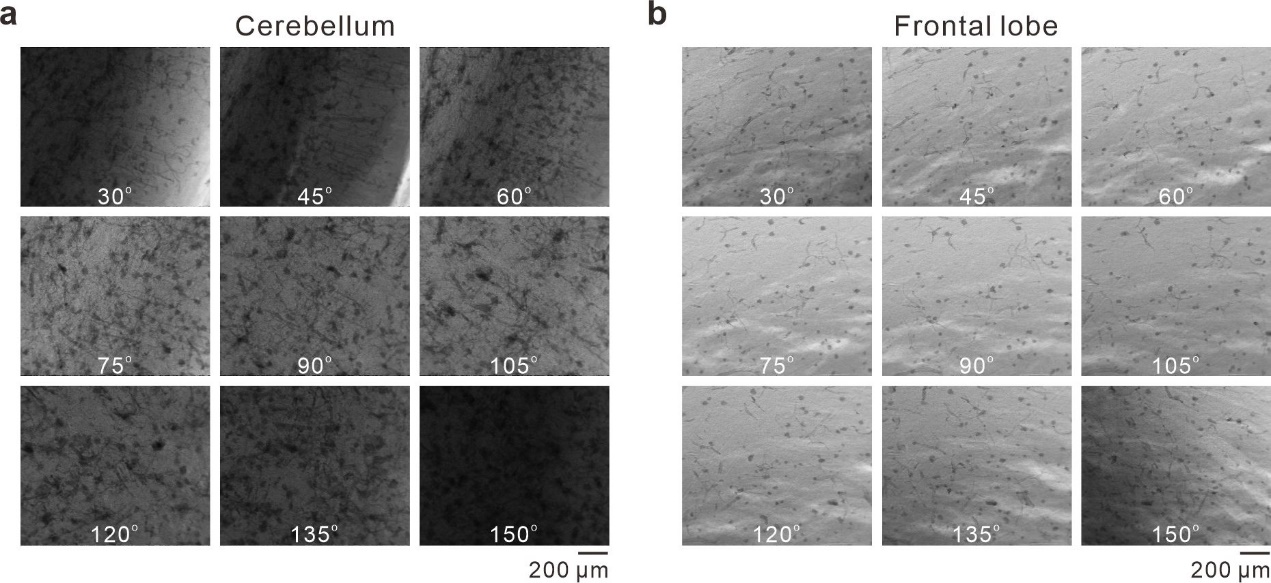


**Figure S6. Projections from different angles. a:** cerebellar tissue, **b:** frontal lobe tissue.

**
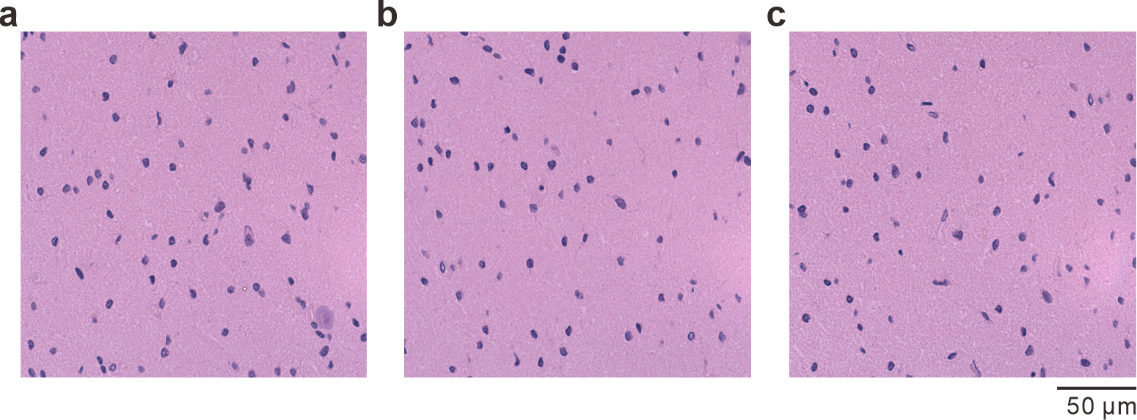
**

**Figure S7. H&E staining results of different human brain samples.** H&E staining results demonstrated that all three groups of human brain tissue samples (**a**: Patient ID 001, **b**: Patient ID 002, and **c**: Patient ID 003) exhibited uniformly stained nuclei and cytoplasm, with no significant tissue damage or alterations in nuclear morphology observed. Scale bar: 50 μm.

**Table S1. Statistics table of stained mean neuron count at different temperature**

| Temperature | Mean neuron count |
| --- | --- |
| 37 ℃ | 4 |
| 26 ℃ | 6 |
| 4 ℃ | 18 |
| NFT | 22 |

**Table S2. Statistics table of stained mean spine count at different temperature**

| Temperature | Mean spine count/  100 μm dendrite |
| --- | --- |
| 37 ℃ | 1 |
| 26 ℃ | 5 |
| 4 ℃ | 8 |
| NFT | 22 |

**Table S3. Statistics table of stained mean neuron count at different staining solution components**

| Component | Mean neuron count |
| --- | --- |
| 1 | 4 |
| 2 | 3 |
| 3 | 5 |
| 4 | 22 |

*1: potassium dichromate and silver nitrate solution; 2: osmium tetroxide, potassium dichromate and silver nitrate solution, 3: glutaraldehyde, potassium dichromate and silver nitrate solution, and 4: potassium dichromate, potassium chromate and mercuric chloride solution.

**Table S4. Statistical table of mean spine count obtained under different staining solution components**

| Component | Mean spine count/  100 μm dendrite |
| --- | --- |
| 1 | 0 |
| 2 | 0 |
| 3 | 0 |
| 4 | 22 |

*1: potassium dichromate and silver nitrate solution; 2: osmium tetroxide, potassium dichromate and silver nitrate solution, 3: glutaraldehyde, potassium dichromate and silver nitrate solution, and 4: potassium dichromate, potassium chromate and mercuric chloride solution.

**Table S5. Summary of sample information for humans**

| Patient ID | Gender | Age  (years) | Couse of death | PMD  (hours) |
| --- | --- | --- | --- | --- |
| 001 | F | 93 | Heart failure | 2 |
| 002 | F | 67 | Terminal rectal cancer | 5 |
| 003 | M | 60 | Bile duct cancer | 20 |
